# Medication adherence and its determinants in patients after myocardial infarction

**DOI:** 10.1038/s41598-020-68915-1

**Published:** 2020-07-21

**Authors:** Łukasz Pietrzykowski, Piotr Michalski, Agata Kosobucka, Michał Kasprzak, Tomasz Fabiszak, Wioleta Stolarek, Jolanta M. Siller-Matula, Aldona Kubica

**Affiliations:** 1 Department of Health Promotion, Collegium Medicum, Nicolaus Copernicus University, Curie Skłodowskiej St. 9, 85-094 Bydgoszcz, Poland; 2 Department of Cardiology and Internal Diseases, Collegium Medicum, Nicolaus Copernicus University, Bydgoszcz, Poland; 3 Department of Pharmacology and Therapeutics, Collegium Medicum, Nicolaus Copernicus University, Bydgoszcz, Poland; 4 Department of Cardiology, Medical University of Vienna, Vienna, Austria

**Keywords:** Cardiology, Risk factors

## Abstract

Non-adherence to prescribed medication is a serious limitation of long-term treatment in patients after myocardial infarction (MI), which can be associated with medical, social and economical consequences. Improvement of medication adherence has been shown to be a challenge for healthcare providers. The aim of this study was to evaluate changes in medication adherence and variability of adherence determinants during follow-up in patients after MI. A single-center, cohort observational study was conducted in 225 post-MI patients treated with primary coronary intervention (PCI) (27% women and 73% men) aged 30–91 years. Adherence was defined as availability of evaluated drugs within 1-year after discharge from hospital, based on completed prescriptions data obtained from the National Health Fund. The analysis of therapeutic plan realization (adherence to medication prescribed at discharge from hospital) embraced only reimbursed drugs: ACEIs (ramipril, perindopril), P2Y12 receptor inhibitors (clopidogrel) and statins (atorvastatin, simvastatin, rosuvastatin). Sufficient adherence was defined as ≥ 80%. During 1-year follow-up, adherence for all three drug classes was 64 ± 25%, with 67 ± 32% for ACEIs, 62 ± 34% for P2Y12 receptor inhibitor and 64 ± 32% for statins. A gradual decline in adherence was observed from 65% ± 26% in the first quarter of follow-up to 51% ± 34% in the last quarter of follow-up (p < 0.00001). Sufficient adherence for all drugs classes was found only in 29% of patients throughout the whole follow-up period (44% for ACEI, 36% for P2Y12 receptor inhibitor and 41% for statins). According to a multivariate analysis, age, prior CABG, level of education, place of residence, economic status and marital status were independent predictors of drug adherence. Whereas patients > 65 years and having a history of prior CABG more often had an insufficient adherence to drugs, married and hypertensive patients, city inhabitants and patients with higher education tended to have a sufficient drug adherence. Adherence to pharmacotherapy after myocardial infarction decreases over time in a similar manner for all pivotal groups of drugs prescribed after MI. A number of socioeconomic and clinical factors have been identified to affect medication adherence over time.

## Introduction

The long-term treatment of patients after myocardial infarction (MI) is based on implementation of a therapeutic plan including lifestyle changes and pharmacotherapy^1–3^. According to the of European Society of Cardiology guidelines for the management of patients with acute myocardial infarction, therapy in this subset of patients includes dual antiplatelet treatment (DAPT) for 12 months, angiotensin-converting enzyme inhibitors (ACEI) or angiotensin receptor blockers (ARB) if ACEI are contraindicated, beta-blockers, and statins^4^. However, data available on patient adherence to the therapeutic plan (medication prescribed at discharge from hospital) raise concern. A meta-analysis by Naderi et al.^5^ including 20 studies and assessing the extent of adherence to coronary heart disease preventive drug therapy, yield an overall adherence of 57% over a median treatment period of 24 months, with 66% for secondary and only as little as 50% for primary prevention studies. Among MI patients in the PREMIER study 68 (13.6%) discontinued antiplatelet therapy with thienopyridine within 30 days after discharge^6^.

Kubica et al.^7^ reported regular intake of clopidogrel according to prescription only in 54.3% of patients during 1-year follow-up after MI^7^, with non-adherence to this therapy resulting in fourfold higher recurrence rate of acute coronary syndrome^8,9^. Factors affecting adherence have been evaluated in numerous previous studies^10–14^, however, inconsistency of results and huge discrepancies of reported adherence level after hospitalization warrant further research. Moreover, previous studies did not differentiate factors that affect the failure to implement the recommended therapy after hospital discharge from ones that affect the discontinuation of chronic therapy. Furthermore, according to our best knowledge, data regarding time related changes in adherence determinants were not previously reported.

Therefore, the aim of this study was to evaluate changes in medication adherence and variability of adherence determinants during follow-up in patients after MI.

## Methods

### Study design and patients

A total of 225 consecutive patients hospitalized for MI between May 2015 and July 2016 at the Department of Cardiology and Internal Medicine of the University Hospital No. 1 in Bydgoszcz, Poland, who met the inclusion criteria were enrolled in this single-center, cohort observational study. The study is part of a major project (the impact of readiness for discharge from hospital and socio-demographic factors on adherence, quality of life, functioning in disease and selected clinical parameters in patients with chronic diseases) approved by the Bioethics Committee of the Nicolaus Copernicus University in Toruń (approval No. KB 312/2015). The following inclusion criteria were applied: age over 18 years, hospitalization due to acute MI treated with primary coronary intervention (PCI), pharmacotherapy including ACEI (ramipril, perindopril), P2Y12 receptor inhibitor (clopidogrel) and statin (atorvastatin, simvastatin, rosuvastatin).

All study participants provided a written informed consent for participation in the study. Exclusion criteria were defined as: presence of contraindications for study medications or other conditions that may cause their temporary or permanent discontinuation. Patients with impaired contact and/or mental disorders or otherwise unable to provide informed consent to participate in the study, family members and collaborators of research team members as well as prisoners were excluded from the study. Following educational measures provided to study participants during hospitalization and explaining the causes of coronary artery disease, its symptoms and treatment a therapeutic plan including pharmacotherapy was established as an effect of comprehensive involvement of the patient and medical professionals. The availability of study medications within 1-year of hospital discharge was established on the basis of National Health Fund data regarding completed prescriptions.

The study population consisted of 225 patients (26.7% women and 73.3% men) aged 30–91 years (mean age 62.9 ± 11.9). Due to incomplete data regarding study medications (lack of data regarding non-reimbursed drugs), the final analysis comprised 210 patients (93.3% of all study participants) receiving ACEI, 194 (86.2%) treated with a P2Y12 receptor inhibitor, and 222 (98.7%) patients on statin. Due to these limitations, a complete analysis for all three groups of study medication was carried out in 180 patients (80.0% of study participants). Shortening of follow-up due to patient death (8 cases—3.6% of the study population) was taken into account during results evaluation.

The level of adherence and proportion of patients with adherence to treatment ≥ 80% were evaluated. Insufficient adherence was defined as < 80%, whereas sufficient adherence ≥ 80%.

A cut-off point of 80% was applied as according to previous publications adherence rate ≥ 80% is necessary to ensure effectiveness of long-term therapy after MI^15–17^. Based on a 1-year follow-up, an analysis was performed for each medication group separately and for all three groups together. For adherence variability assessment, the follow-up period was split into quarters. Also, influence of selected socio-demographic and clinical factors on adherence to treatment was verified, including age, sex, level of education, employment, economic status, place of residence, marital status, previous diagnosis of coronary artery disease (CAD), previous MI, PCI or coronary artery by-pass graft (CABG), previously diagnosed hypertension, diabetes mellitus and smoking.

### Statistical analysis

Statistical analysis was performed using the Statistica 12.0 package (StatSoft, Tulsa, USA). Continuous variables were presented as medians with interquarter ranges, means with standard deviations and percentages. The Shapiro–Wilk test was applied to evaluate distribution of continuous variables. For quantitative variables with non-normal distribution, non-parametric tests were used. Pairwise deletion method was used for missing data. For comparison of quantitative dependent variables, Friedman rank analysis of variance for repeated measurements and Wilcoxon pairs order test were used. Comparisons between two groups were performed with the Mann–Whitney unpaired rank sum test. For comparisons between three or more groups, the Kruskal–Wallis one-way analysis of variance was used. Comparisons between dependent qualitative variables were performed using the Q-Cochran test and the McNemar test. Categorical variables were compared using the χ^2^ test with the Yates’ correction if required. Differences were considered significant at p < 0.05. p values in the range of ≥ 0.05 and < 0.10 were considered a trend towards statistical significance, while p ≥ 0.10 was regarded as statistically insignificant. Multivariate analysis indicating independent factors influencing adherence was carried out with multiple regression and multivariate logistic regression analysis. In order to select the best regression models, the stepwise backward regression method was applied. Initially, parameters with p < 0.1 in the univariate analysis were introduced to the model, then statistically insignificant variables (p ≥ 0.05) were subsequently removed from the multivariate model according to the decreasing p value.

### Ethical approval

This article does not contain any studies with animals performed by any of the authors. All procedures performed in studies involving human participants were in accordance with the ethical standards of the institutional and/or national research committee and with the 1964 Helsinki declaration and its later amendments or comparable ethical standards.

### Informed consent

Informed consent was obtained from all individual participants included in the study.

## Results

### Level of adherence to treatment

**Figure 1:**
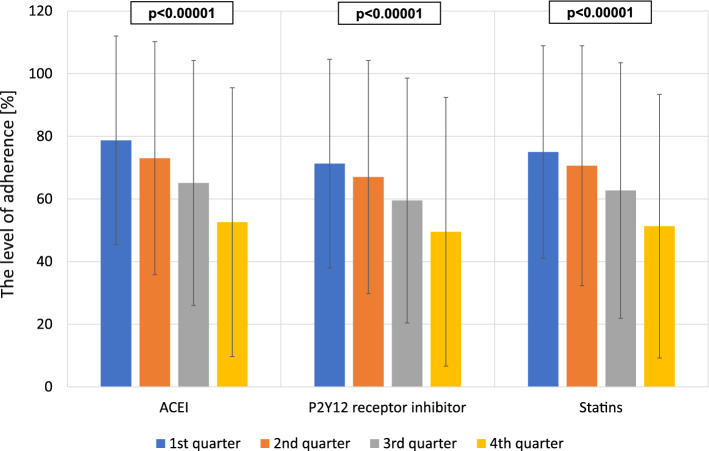
The level of adherence to treatment expressed as drug availability (number of tablets/number of days) in consecutive follow-up quarters.

The mean adherence level during 1-year of follow-up for all three groups of medications was 64.1 ± 24.5%, with a value of 67.2 ± 31.8% for ACEI, 61.6 ± 34.2% for P2Y12 receptor inhibitors, and 64.4 ± 32.1% for statins. Over time, a gradual decline in adherence was observed for all groups of medications (Fig. 1). A combined analysis for all three groups of medications showed a decrease in the level of adherence from 65.0 ± 25.8% in the first quarter down to 50.7 ± 34.4% in the last quarter of follow-up (p < 0.00001).

### Proportion of patients with sufficient drug adherence

**Figure 2:**
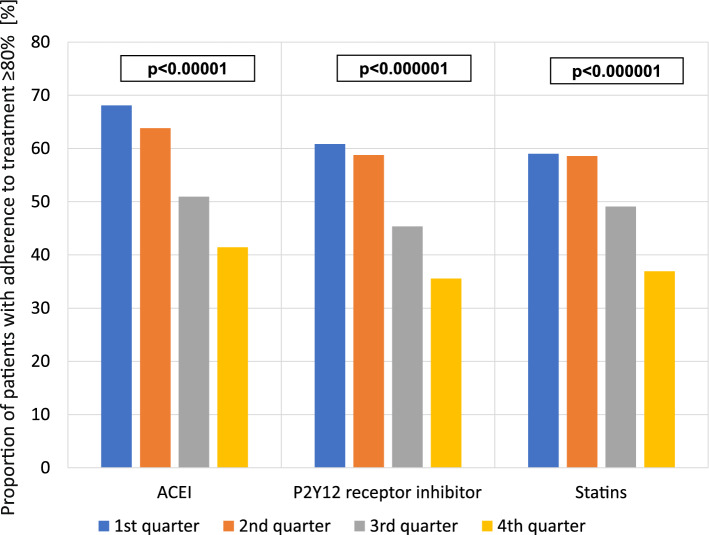
Proportion of patients with adherence to treatment ≥ 80% in consecutive follow-up quarters.

Sufficient adherence for all medication groups was found in 29.4% of patients throughout the whole follow-up period (44.3% for ACEI, 36.1% for P2Y12 receptor inhibitor and 40.9% for statins). A significant decrease in the percentage of patients with adherence ≥ 80% was observed for all three groups of medications in the third and fourth quarter in comparison with a previous quarter of follow-up, while the differences between the first and second quarter were not significant (Fig. 2, Table 1).

**Table 1:** Number of patients with ≥ 80% adherence to treatment with all groups of medications in consecutive follow-up quarters.

Adherence ≥ 80%	1st quarter		2nd quarter		3rd quarter		4th quarter		p
	n	%	n	%	n	%	n	%	
For all three groups of medications	66	36.7	56	31.1	41	22.8	30	16.7	< 0.000001
For none of the three groups of medications	29	16.1	28	15.6	52	28.9	72	40.0	< 0.000001

### Socio-demographic and clinical factors affecting adherence in consecutive quarters of follow-up

Socio-demographic and clinical characteristics of the study group as well as the mean annual level of adherence for individual groups of medications with regard to different factors are presented in Table 2. Tables 3 and 4 present the results of univariate and multifactorial analysis of variance of determinants affecting adherence in annual follow-up and in individual quarters. The relationship between the assessed factors and the level of adherence was mainly observed for ACEI. The results of univariate analysis of determinants affecting adherence ≥ 80% are shown in Table 5.

**Table 2:** Study population characteristics and impact of socio-demographic and clinical factors on adherence in 1-year follow-up.

Parameter	Variable	Total sample		Adherence in 1-year follow-up					
				ACEI		P2Y12 receptor inhibitor		Statins	
		n	%	%	p	%	p	%	p
Gender	Female	60	26.7	64.9 (± 31.8)	0.4952	66.7 (± 29.7)	0.4068	56.5 (± 40.9)	0.2566
	Male	165	73.3	68.0 (± 31.9)		69.7 (± 35.6)		49.4 (± 42.6)	
Age	< 65	129	57.3	**71.5 (± 31.0)**	**0.0071**	61.3 (± 36.3)	0.6520	64.6 (± 34.2)	0.4936
	≥ 65	96	42.7	**61.2 (± 32.1)**		61.9 (± 31.5)		65.3 (± 29.2)	
Employment status	Employed	93	41.3	**74.2 (± 30.3)**	**0.0023**	60.8 (± 37.3)	0.6296	64.6 (± 36.3)	0.6445
	Unemployed	13	5.8	**46.2 (± 39.3)**		70.4 (± 28.2)		69.9 (± 30.8)	
	Old age pensioner	91	40.4	**61.0 (± 31.2)**		60.3 (± 31.0)		63.4 (± 28.2)	
	Disability living allowance recipient	28	12.4	**73.0 (± 28.2)**		63.2 (± 37.9)		64.9 (± 31.3)	
Education	Primary	30	13.3	53.9 (± 38.6)	0.1554	66.0 (± 30.8)	0.788	62.1 (± 34.1)	0.9679
	Vocational	83	36.9	65.0 (± 32.8)		62.1 (± 32.0)		64.3 (± 31.6)	
	Secondary	82	36.4	73.7 (± 26.2)		60.9 (± 36.8)		65.5 (± 31.1)	
	Higher	30	13.3	67.6 (± 33.5)		56.1 (± 38.1)		64.1 (± 35.3)	
Economic status	Very good	14	6.2	53.4 (± 37.3)	0.1129	44.4 (± 37.0)	0.2462	53.4 (± 38.9)	0.4821
	Satisfactory	199	88.4	69.1 (± 30.7)		62.0 (± 34.3)		64.9 (± 31.7)	
	Bad	12	5.3	52.9 (± 38.5)		68.3 (± 26.2)		67.8 (± 32.6)	
	Very bad	0	0	0		0		0	
Marital status	Unmarried	25	11.1	**53.9 (± 36.2)**	**0.0244**	62.7 (± 30.2)	0.8057	54.9 (± 36.4)	0.3877
	Widowed	33	14.7	**60.4 (± 34.3)**		62.7 (± 37.5)		64.9 (± 30.7)	
	Married	167	74.2	**70.4 (± 30.2)**		61.1 (± 34.3)		65.8 (± 31.6)	
Place of residence	City	161	71.6	66.7 (± 32.3)	0.7474	**64.6 (± 33.5)**	**0.0438**	64.0 (± 31.1)	0.4810
	The country	64	28.4	68.4 (± 31.5)		**54.2 (± 35.1)**		65.7 (± 34.8)	
History of CAD	Yes	102	45.3	64.1 (± 32.3)	0.2751	60.2 (± 34.6)	0.7585	61.8 (± 33.9)	0.6201
	No	123	54.7	69.0 (± 31.5)		62.3 (± 34.1)		65.9 (± 31.1)	
Prior MI	Yes	64	28.4	**58.3 (± 31.8)**	**0.0050**	57.5 (± 34.5)	0.2637	**58.3 (± 31.8)**	**0.0016**
	No	161	71.6	**70.9 (± 31.2)**		63.3 (± 34.1)		**70.9 (± 31.2)**	
Prior PTCA	Yes	82	36.4	**61.6 (± 32.1)**	**0.0210**	56.6 (± 34.2)	0.1114	51.2 (± 43.1)	0.8148
	No	143	63.6	**70.7 (± 31.3)**		64.3 (± 34.0)		51.4 (± 41.7)	
Prior CABG	Yes	34	15.1	57.2 (± 33.6)	0.0543	**46.9 (± 34.1)**	**0.0105**	61.6 (± 34.6)	0.7224
	No	191	84.9	69.0 (± 31.2)		**64.1 (± 33.7)**		65.0 (± 31.7)	
Hyperlipidemia	Yes	151	67.6	65.5 (± 31.2)	0.1211	**57.9 (± 34.7)**	**0.0313**	63.9 (± 31.9)	0.6007
	No	73	32.4	70.6 (± 33.1)		**68.8 (± 32.3)**		65.6 (± 32.7)	
Diabetes	Yes	63	28.0	68.2 (± 31.4)	0.7770	64.7 (± 33.0)	0.5150	63.5 (± 33.2)	0.9160
	No	157	71.0	66.8 (± 32.1)		60.5 (± 34.6)		64.8 (± 31.8)	
Hypertension	Yes	165	73.3	67.4 (± 32.1)	0.8188	59.3 (± 35.0)	0.1763	64.5 (± 31.8)	0.9906
	No	60	26.7.0	66.5 (± 31.4)		67.4 (31.6)		63.3 (± 33.2)	
Smoking status (current)	Yes	85	37.8	64.8 (± 31.8)	0.3086	63.5 (± 33.6)	0.4828	61.0 (± 33.0)	0.1993
	No	140	62.2	68.7 (± 31.9)		60.3 (± 34.7)		66.6 (± 31.5)	

*CAD* coronary artery disease, *MI* myocardial infarction, *PTCA* percutaneous transluminal coronary angioplasty, *CABG* coronary artery by-pass graft.

Bold values indicate p < 0.05.

**Table 3:** Determinants of adherence according to groups of medications and periods of follow-up.

Medications	Follow-up period				
	1st quarter	2nd quarter	3rd quarter	4th quarter	1-year
**Factors associated with the increase in adherence**					
ACEI	Employment status—employed^a^ Marital status—married^a^	Employment status—employed^a^ Education—secondary or higher^a^ Marital status—married^a^ Economic status—very good and satisfactory^b^	Employment status—employed^a^ Education—secondary or higher^a^ Marital status—married^a^ Economic status—very good and satisfactory^a^	Employment status—employed^a^ Marital status—married^a^ Education—secondary or higher^b^ Family burden^b^	Employment status—employed^a^ Marital status—married^a^ Education—secondary or higher^b^
P2Y12 receptor inhibitor					
Statins	Employment status—employed^b^	Marital status—married^a^			
**Factors associated with the decrease in adherence**					
ACEI	Age ≥ 65^a^ Prior PTCA^a^ Prior CABG^a^ Prior MI^b^	Age ≥ 65^a^ Prior MI^a^ Prior PTCA^a^	Age ≥ 65^a^ Prior MI^a^ Hipierlipidemia ^a^		Prior PTCA^a^ Prior MI^a^ Prior CABG^b^
P2Y12 receptor inhibitor	Prior PTCA^a^ Prior CABG^a^	Prior PTCA^a^ Prior CABG^a^ Hyperlipidemia^b^	Hipierlipidemia^a^ Prior CABG^b^ Smoking^b^	Prior CABG^b^	Prior MI^a^ Prior CABG^a^ Hiperlipidemia^a^
Statins	Prior MI^a^ Prior PTCA^a^ History of CAD^a^ Age ≥ 65^b^	Prior MI^a^ Prior PTCA^a^ History of CAD^a^	Prior MI^a^ Living status—with family^b^	Smoking^b^	Prior MI^a^
ACEI + P2Y12 receptor inhibitor + Statins	Prior MI^a^ Prior PTCA^a^ Prior CABG^a^ Economic status—very good^b^	Prior MI^a^ Prior PTCA^a^ Prior CABG^a^ Hipierlipidemia^a^ Economic status—very good^b^	Prior MI^a^ Hipierlipidemia^a^ Economic status—very good^a^ Smoking^b^ Prior PTCA^b^		Prior MI^a^ Prior PTCA^a^ Prior CABG^a^ Hiperlipidemia^a^ Economic status—very good^b^

Variables with p ≥ 0.1 are not presented.

*CAD* coronary artery disease, *MI* myocardial infarction, *PTCA* percutaneous transluminal coronary angioplasty, *CABG* coronary artery by-pass graft.

^a^p < 0.05

^b^0.05 ≤ p < 0.1

**Table 4:** Multivariate analysis of determinants affecting adherence according to groups of medications and periods of follow-up.

Medications	Follow-up period	Determinants	β coefficient	p
ACEI	1st quarter	Marital status—married	0.1773 (± 0.0676)	0.0093
		Prior CABG	− 0.1564 (± 0.0676)	0.0216
	2nd quarter	Education—secondary or higher	0.1652 (± 0.0666)	0.0139
		Marital status—married	0.1820 (± 0.0666)	0.0068
		Prior MI	− 0.1785 (± 0.0666)	0.0079
	3rd quarter	Education—secondary or higher	0.1878 (± 0.0672)	0.0057
		Marital status—married	0.1485 (± 0.0669)	0.0276
		Prior MI	− 0.1899 (± 0.0672)	0.0052
	4th quarter	Employment status—employed	0.1723 (± 0.0692)	0.0136
		Family burden	0.1385 (± 0.0692)	0.0468
	1-year	Marital status—married	0.1717 (± 0.0666)	0.0104
		Prior MI	− 0.1971 (± 0.0666)	0.0035
		Education—secondary or higher	0.1790 (± 0.0667)	0.0079
P2Y12 receptor inhibitor	2nd quarter	Prior CABG	0.1525 (± 0.0713)	0.0486
			− 0.1533 (± 0.0713)	0.0328
	3rd quarter	Hiperlipidemia	0.1778 (± 0.0705)	0.0125
			− 0.1945 (± 0.0705)	0.0063
	1-year	Prior CABG	0.1485 (± 0.0704)	0.0362
			− 0.1878 (± 0.0704)	0.0083
ACEI + P2Y12 receptor inhibitor + Statins	2nd quarter	Prior MI	− 0.2349 (± 0.0730)	0.0015
		Economic status—very good	− 0.1449 (± 0.0730)	0.0486
	3rd quarter	Prior MI	− 0.1742 (± 0.0737)	0.0192
		Economic status—very good	− 0.1814 (± 0.0737)	0.0148
	1-year	Prior MI	− 0.2398 (± 0.0726)	0.0011
		Economic status—very good	− 0.1544 (± 0.0726)	0.0349

Only variables with p < 0.05 are presented.

*MI* myocardial infarction, *CABG* coronary artery by-pass graft.

**Table 5:** Determinants affecting proportion of patients with adherence ≥ 80% according to groups of medications and periods of follow-up.

Medications	Follow-up period				
	1st quarter	2nd quarter	3rd quarter	4th quarter	1-year
**Factors associated with the increase in the proportion of patients with adherence ≥ 80%**					
ACEI	Marital status—married^a^ Hypertension^b^	Employment status—employed^a^ Marital status—married^a^ Economic status—very good and satisfactory^b^	Employment status—employed^a^ Marital status—married^b^	Employment status—employed^a^	Employment status—employed^a^
P2Y12 receptor inhibitor	Education—primary^b^				
Statins	Marital status—married^a^ History of CAD^a^	Marital status—married^b^			
**Factors associated with the decrease in the proportion of patients with adherence ≥ 80%**					
ACEI	Age ≥ 65^a^ Prior CABG^a^	Age ≥ 65^a^ Education—primary^a^	Age ≥ 65^a^ Economic status—very good^a^ Hyperlipidemia^a^ Education—primary^b^	Age ≥ 65^a^	Age ≥ 65^a^
P2Y12 receptor inhibitor	Age ≥ 65^b^ Prior CABG^b^	Prior CABG^a^	Hyperlipidemia^a^ Living status—with family^b^		Prior CABG^a^
Statins	Age ≥ 65^a^	History of CAD^a^			
ACEI + P2Y12 receptor inhibitor + Statins	Prior CABG^b^	Prior CABG^a^ Economic status—very good^b^ Education—primary^b^	Economic status—very good^a^ Hyperlipidemia^a^	Smoking^b^ Economic status—very good^b^	Economic status—very good^a^ Hyperlipidemia^a^ Prior CABG^b^

Variables with p ≥ 0.1 are not presented.

*CAD* coronary artery disease, *CABG* coronary artery by-pass graft.

^a^p < 0.05

^b^0.05 ≤ p < 0.1

### Socio-demographic factors affecting adherence in consecutive quarters of follow-up

Impact of different socio-demographic factors on adherence was mainly observed with ACEI.

#### Age

Patients below 65 years of age were characterized by significantly higher adherence to treatment with ACEI in comparison with older patients in quarters 1–3 of follow-up, with the highest difference observed in the 3rd quarter: 70.3 ± 39.1% vs. 57.6 ± 38.0%, p = 0.0033. Younger patients demonstrated a higher prevalence of annual adherence levels ≥ 80% for ACEI (51.6% vs. 34.1% p = 0.0115) as well as separately in each of the quarters of follow-up with the highest difference in the 3rd quarter: 59.0% vs. 40.7%, p = 0.0092.

A higher proportion of patients with adherence ≥ 80% for statins was also observed in younger vs. older patients in the 1st quarter of follow-up: 65.3% vs 50.5%, p = 0.0262.

#### Employment status

Patient employment status was also found to have impact on adherence to treatment with ACEI in all consecutive quarters of follow-up. Adherence was significantly higher in employed patients in comparison to pensioners with the highest difference in the 3rd quarter of follow-up (74.2 ± 37.3% vs. 58.1 ± 38.3%, p = 0.0034). A higher proportion of patients with adherence to ACEI treatment ≥ 80% was also observed in employed patients in comparison to the unemployed and pensioners in 1-year follow-up (56.1% vs. 35.5%; p = 0.0029). The highest difference was observed in the 3rd quarter (62.9% vs. 42.8%, p = 0.0042).

#### Education status

Secondary and higher education were associated with higher adherence to treatment with ACEI in comparison to patients with primary or vocational education in ^the 2nd and 3rd quarter^ of follow-up reaching a difference of 71.7 ± 36.4% vs. 58.8 ± 40.6%, p = 0.0148 in the 3rd quarter. The proportion of patients with adherence to treatment with ACEI ≥ 80% increased along with the level of education (primary 44.0%, vocational 62.9%, secondary 69.7%, higher 70,1%; p = 0.0349 for trend).

#### Marital status

A higher mean level of annual adherence to treatment with ACEI (p = 0.0244) was also found in married patients (70.4 ± 30.2%), compared with the unmarried (53.9 ± 36.2%) and widowed (60.4 ± 34.3%). Similar differences between married vs. unmarried/widowed patients were found in first three consecutive quarters of follow-up with the highest difference in the 2nd quarter: 76.9 ± 35.3% vs. 61.5 ± 40.5%, p = 0.046. As a result, there was a higher proportion of married patients, in comparison to unmarried and widowed study participants, with ≥ 80% adherence to treatment with ACEI in the 1st and 2nd quarters of follow-up. The difference was most prenounced in the 1st quarter: 73.1% vs. 53.7%, p = 0.0085.

With regard to statins, the level of adherence was higher in married patients compared with unmarried and widowed in the 2nd quarter (73.5 ± 37.3% vs. 62.1 ± 40.4%, p = 0.0401). The percentage of patients with ≥ 80% adherence to treatment with statins was higher (p = 0.0405) among married (62.1%) and widowed patients (57.5%) than in unmarried patients (40.0%).

Adherence to treatment with ACEI in the 3rd quarter was also higher (p = 0.0379) in study participants declaring their economic status as very good or satisfactory (66.6 ± 30.2%) compared with those who perceived their status as bad or very bad (43.7 ± 43.5%). The percentage of patients with adherence ≥ 80% differed with regard to economic status in the 3rd quarter for ACEI (very good 18.1%, satisfactory 54.6%, poor 35.7%; p = 0.00301) and in the whole 1-year follow-up for each of the three medication groups (very good 0%, satisfactory 35.0%, poor 16.6%; p = 0.0452).

#### Place of accommodation

The analysis of treatment with P2Y12 receptor inhibitor revealed a higher level of adherence (p = 0.01270) in city inhabitants (64.2 ± 39.7%), compared with people living in the countryside (48.4 ± 39.8%) for the entire annual follow-up. Consequently, adherence ≥ 80% was more commonly encountered among city inhabitants than in rural area residents (40.8% vs. 24.5%, p = 0.0311). Similar results were obtained regarding proportion of patients with ≥ 80% adherence to ACEI in the 1st and 3rd quarters of follow-up, with the highest difference in the 3rd quarter: 52.2% vs. 32.1% p = 0.0113.

### Clinical factors affecting adherence in consecutive quarters of follow-up

Clinical factors influenced to a different extent the level of adherence to treatment within all analyzed groups of medications.

Clinical factors such as CAD diagnosed before admission, previous MI, PCI, CABG and hyperlipidemia predisposed to lower adherence to medical treatment.

Adherence to medication with statins in the 1st and 2nd quarter of follow-up was lower in patients with CAD diagnosed before admission in comparison with those without such diagnosis. The difference was biggest in the 2nd quarter: 64.0 ± 40.8% vs 75.8 ± 35.6%, p = 0.0229. As a result, patients with a prior diagnosis of CAD presented with a lower proportion of adherence ≥ 80% in the 1st and 2nd quarters with the highest difference in the 1st quarter: 51.0% vs. 65.6% p = 0.0280.

Adherence rates in the 1st, 2nd and 3rd quarters of follow-up were also lower in patients with a prior myocardial infarction in contrast to those with no history of heart attack before index hospitalization. The highest difference for ACEI was observed in the 3rd quarter: 54.4 ± 39.3% vs. 69.4 ± 38.2%, p = 0.0080, and for statins in the 2nd quarter: 58.7 ± 42.4% vs. 74.9 ± 35.9%, p = 0.0119.

Also, previous PTCA treatment had a distinct impact on adherence to medication. In patients without prior PTCA, the level of adherence was higher than in those after a prior PTCA in the 1st and 2nd quarter of follow-up for all monitored medication groups, whether analyzed separately or together. The difference was highest for ACEI in the 2nd quarter of follow-up: 78.2 ± 34.6% vs. 64.8 ± 39.8%, p = 0.0067; for P2Y12 receptor inhibitor in the 1st quarter: 75.3 ± 36.5% vs. 64.1 ± 39.8%, p = 0.0209; and for statin in the 2nd quarter: 74.4 ± 36.8% vs. 63.8 ± 40.3%, p = 0.05.

Differences in levels of adherence were also found in the 1st and 2nd quarter of follow-up regarding previous CABG status. Presence of a history of CABG was associated with a lower adherence to treatment with P2Y12 receptor inhibitor with the highest difference in the 2nd quarter of follow-up: 69.5 ± 39.6% vs. 53.1 ± 41.3%, p = 0.0127).

Patients with previous CABG less frequently achieved adherence ≥ 80% comparing to others: for ACEI in the 1st quarter of follow-up (51.5% vs. 71.1%, p = 0.0260), for P2Y12 receptor inhibitor in the entire follow-up (13.8% vs. 40.0%, p = 0.0067), and in the 2nd quarter (41.3% vs. 62.5%, p = 0.0339).

Presence of priorly diagnosed hyperlipidaemia was associated with higher adherence in the 3rd quarter of follow-up for ACEI (71.0 ± 40.1% vs.62.1 ± 38.2%, p = 0.0313) and P2Y12 receptor inhibitor (70.4 ± 38.5% vs. 53.9 ± 40.1%, p = 0.0045). Consequently, the prevalence of adherence ≥ 80% in this subset of patients was lower in the 3rd quarter for ACEI (46.3% vs. 61.4%, p = 0.0401), and for P2Y12 receptor inhibitors.

### Multivariate analysis of adherence determinants

The results of multivariate analysis of determinants affecting adherence ≥ 80% are shown in Table 6.

**Table 6:** Multivariate analysis of determinants affecting proportion of patients with adherence ≥ 80% according to groups of medications and periods of follow-up.

Medications	Follow-up period	Determinants	OR (95% Cl)	p
ACEI	1st quarter	Age ≥ 65	0.524 (0.280–0.984)	0.0443
		Marital status—married	2.175 (1.116–4.239)	0.0225
		Prior CABG	0.339 (0.150–0.766)	0.0093
		Hypertension	2.411 (1.186–4.901)	0.0151
P2Y12 receptor inhibitor	1st quarter	Age ≥ 65	0.546 (0.298–0.999)	0.0496
		Education (for every degree of better education on the 4-degree scale: primary, vocational, secondary, higher)	0.612 (0.425–0.882)	0.0084
		Place of residence^a^ (for each degree on the 3-degree scale: the country, town, city)	0.651 (0.455–0.930)	0.0184
		Prior CABG	0.414 (0.181–0.948)	0.0369
	2nd quarter	Place of residence (for each degree on the 3-degree scale: the country, town, city)	0.645 (0.454–0.915)	0.0139
		Hyperlipidemia	0.341 (0.181–0.642)	0.0009
	1-year	Prior CABG	0.223 (0.073–0.675)	0.0080
		Place of residence—city	2.281 (1.127–4.614)	0.0218
Statins	1st quarter	Age ≥ 65	0.565 (0.326–0.979)	0.0418
		History of CAD	0.571 (0.330–0.987)	0.0446
ACEI + P2Y12 receptor inhibitor + Statins	2nd quarter	Education (for every degree of better education on the 4-degree scale: primary, vocational, secondary, higher)	1.584 (1.072–2.340)	0.0209
		Economic status—very good	0.082 (0.010–0.711)	0.0232
		Prior CABG	0.177 (0.058–0.544)	0.0025

City > 100,000 inhabitants; Town ≤ 100,000 inhabitants.

Only variables with p < 0.05 are presented.

*CAD* coronary artery disease, *CABG* coronary artery by-pass graft.

Age over 65 almost doubles the probability of low adherence (< 80%) in the first quarter of follow-up for ACEI, P2Y12 receptor inhibitor and statin. Prior CABG procedure was associated with a 2.9-fold and 2.4-fold reduction in the probability of adherence ≥ 80% in the 1st quarter of follow up for ACEI and for P2Y12 receptor inhibitor, respectively. For all medication groups evaluated together the magnitude of the reduction was even higher: 4.5-fold for the complete annual follow-up and 5.0-fold in the 2nd quarter. The level of education and place of residence were also associated with the prevalence of ≥ 80% adherence to P2Y12 receptor inhibitor—in the 1st quarter of follow-up for the for the former, and in the 1st and 2nd quarters for the latter. Moreover, economic status had a strong impact on adherence to medication with all assessed drugs in the 2nd quarter. Higher probability of adherence ≥ 80% was found for ACEI in married patients and those with hypertension, in the 1st quarter of follow-up, for P2Y12 receptor inhibitor in city inhabitants, and for all evaluated groups of medications in patients with higher education.

## Discussion

According to our best knowledge, it is the first study evaluating not only the level of adherence to medication and its determinants, but also the variability of these determinants during the follow-up after hospital discharge.

Moreover, the analysis of time related decrease of adherence and factors influencing adherence was performed separately for three pivotal groups of drugs recommended after MI. We have also revealed that factors responsible for lack of implementation of the recommended medications (adherence < 80% in the 1st quarter of follow-up) into daily therapy after discharge from hospital (25% of patients) are different in comparison to factors associated with premature cessation of maintenance medication occurring with increasing frequency in subsequent quarters of follow-up: 2nd—6.7%, 3rd—11.9%, and 4th—17.8%.

The average level of adherence during the 1-year follow-up for all 3 groups of medications combined together was 64.1 ± 24.5%, with similar results for each of them individually. These findings are in line with those obtained by Naderiet et al.^5^ in a meta-analysis of 20 studies evaluating 7 groups of drugs, with a mean adherence level of 57.0%. They also align with 68.6% adherence for statins and 66.4% adherence for ACEI/ARB reported by Choudhryet et al.^18^. Similarly to Narderi et al.^5^, we observed an adherence decline for all three groups of medications over consecutive quarters of follow-up. When evaluating implementation of prescriptions for dual antiplatelet therapy, Thim et al.^19^ noticed a significant decrease in adherence starting after 3–4 months of the therapy.

The level of adherence ≥ 80% is generally considered indispensable for the effectiveness of long-term therapy^15–17^. According to our study, such level of adherence is present only in 44.3% of patients for ACEIs, 36.1% for P2Y12 receptor inhibitor, and 40.9% for statins during 1 year of follow-up. Analogeous results in the Hungarian population, as reported by Jánosi^20^ were: 64.0% for ARB/ACEI, 54.4% for P2Y12 receptor inhibitors and 64.9% for statins. It should be noted however, that the study by Jánosi included only patients without reinfarction or death within 180 days from the initial cardiovascular event. In a study published by Kirchmayer et al.^21^, in the Italian population adherence level ≥ 80% was observed in 64.4% of patients for ARB/ACEI, 81.9% for P2Y12 receptor inhibitors and 76.1% for statins. Finally, Zhu^16^ reported adherence ≥ 80% with regard to treatment with P2Y12 receptor inhibitors in 66.8% of patients.

Our analysis of adherence alterations showed a gradual decrease in the proportion of patients with adherence level ≥ 80% in consecutive quarters of follow-up. The greatest decrease was observed in the 3rd and 4th quarters for each of the investigated groups of medications. Korhonen et al.^22^ observed the level of adherence to treatment ≥ 80% in patients after myocardial infarction to be 49% for ARB/ACEI, beta-blockers and statins. Mathews^23^ reported that within 6 months of therapy, 31% of patients discontinued their medication with at least one of the following: ARB/ACEI, aspirin, statin, beta-blocker or P2Y12 receptor inhibitor.

Searching for determinants of non-adherence to treatment we additionally performed an analysis of results in sub-groups defined according to socio-demographic (sex, age, level of education, employment, economic status, place of residence, marital status) and clinical factors (previous diagnosis of CAD, previous MI, PCI or CABG, previously diagnosed hypertension, diabetes mellitus and smoking) in consecutive quarters of follow-up.

According to multivariate analysis some of these factors are associated with implementation and/or continuation of prescribed medication.

Similarly to a study by Wonga et al.^24^ we did not find gender to affect the level of adherence. However, the results of other studies are inconsistent, some of them suggesting predisposition of women to lower^21,25^ or to higher^26^ level of adherence to pharmacotherapy.

We have shown that age influences adherence to treatment. Younger patients (under 65 years of age) were more prone to implement medication with ACEI, P2Y12 receptor inhibitor and statin and to continue treatment with ACEI throughout the entire follow-up period as well as in the 1st, 2nd and 3rd quarter. Patients < 65 years old adhered better to medication. Zhu et al.^16^ also observed that younger age is associated with higher adherence to treatment with P2Y12 receptor inhibitors. Nevertheless, in some studies lower adherence was observed in younger patients^16,27,28^ while other studies suggest that age does not affect the level of adherence to treatment^5,24^.

With regard to the level of education, we observed better adherence to treatment with P2Y12 receptor inhibitor, ACEI and statin in patients with higher level of education immediately after discharge from the hospital. These observations are in line with previously published reports by Ho et al.^8^ and by Crowley et al.^27^ showing association between higher level of education and higher level of adherence to medication.

We found unemployed patients to be of lower adherence with regard to treatment with ACEI during the whole follow-up period as well as in all consecutive quarters. The association between socio-economic factors including employment status and adherence to medication was reported in numerous publications, however the results again are inconsistent^10,24,27,29^. Our observations suggest that living in rural areas is associated with higher risk of non-implementation and discontinuation of treatment with P2Y12 receptor inhibitor. According to our knowledge, no reports regarding this issue have been published so far.

Marriage as a factor improving adherence to therapeutic recommendations regarding measurements of blood pressure has been shown by Kanga et al.^30^. Similar observations were also reported by Ho et al.^8^ and by Crowley et al.^27^. We confirmed these observations showing a higher probability of implementation of treatment with ACEI in married patients.

It could be expected that a previously diagnosed disease or previously introduced treatment can improve adherence to new treatment, however in our study previous MI, PTCA, CABG and hyperlipidaemia were associated with a lower level of adherence to treatment mainly with ACEI and P2Y12 receptor inhibitor. The multivariate analysis demonstrated an association between previous CABG and low adherence to treatment with pivotal drugs in patients after MI. We cannot offer any reliable explanation for this fact, as we did not follow all patients after CABG, but only those who experienced MI after surgery, therefore, our observations cannot be generalized to all patients who have undergone surgical revascularization. On the other hand, discontinuation of ACEI and P2Y12 receptor inhibitor after MI may have a much stronger impact on clinical outcome in CABG patients compared with general population due to the presence of diffuse atherosclerosis in the former. Accordingly, it may be assumed that previous CABG itself does not predispose to lower adherence, but low adherence in CABG patients is associated with a particularly high risk of MI. Previous non-adherence was shown to be one of risk factors of low adherence^31^. Therefore, it is quite likely that the study population mainly contained those patients after CABG in whom low adherence led to MI.

Regardless of our explanation, in some previously published studies, higher adherence was observed in patients without heart disease burden^10,16,32^. These results suggest that people with previous cardiovascular events still do not sufficiently follow therapeutic recommendations. Low adherence to treatment in patients after MI may be caused by fear of therapy side effects, lack of information regarding the disease and therapy, and economic factors^14,21,23,33,34^.

Summing up, improvement of the medication adherence is a challenging task. We have previously reported that low adherence in patients after MI was associated with four-fold higher risk of acute coronary syndrome and two-fold higher risk of non-scheduled cardiovascular hospitalization^7^. The results of our study indicate two important issues to be addressed: (1) lack of implementation of the therapy recommended at discharge from hospital by a significant percentage of patients, (2) progressive decline in adherence to treatment from one quarter to another. Identifying the factors responsible for the decline in adherence in the sequential quarters helps better understand the mechanisms governing this phenomenon and apply targeted corrective interventions. Additional educational and motivational efforts should be directed to elderly, less educated, living alone patients and those after CABG in order to increase the likelihood of implementation of prescribed medication after discharge from hospital. Maintenance of medication during long term treatment requires special support in rural residents and patients with lower economic status.

## Limitations of the study

The data retrieved from the National Health Fund are limited only to medicines covered by the reimbursement program. Therefore, B-blockers, ARBs and new P2Y12 receptor inhibitors (ticagrelor and prasugrel) were not analyzed. Also, we did not obtain data for aspirin since this drug is available without prescription. Thus, only patients receiving reimbursed drugs were included in the study. Moreover, time distribution of drug availability as an indirect method of adherence evaluation is burdened by a bias that is difficult to estimate. Nevertheless, this method was commonly used in similar studies rendering our results comparable. The possibility of feedback from patients regarding the causes of therapy discontinuation was limited due to study protocol.

## Conclusion

Adherence to pharmacotherapy after myocardial infarction decreases over time in similar manner for all pivotal groups of drugs prescribed after MI. A number of socioeconomic and clinical factors have been identified to affect medication adherence over time.

## References

[1] Kosobucka A (2018). Adherence to treatment assessed with the Adherence in Chronic Diseases Scale in patients after myocardial infarction. Patient Prefer. Adher..

[2] Nieuwlaat R (2014). Interventions for enhancing medication adherence. Cochrane Database Syst. Rev..

[3] Kubica A (2017). The Adherence in Chronic Diseases Scale—a new tool to monitor implementation of a treatment plan. Folia Cardiol..

[4] Ibanez A, ESC Scientific Document Group (2018). 2017 ESC Guidelines for the management of acute myocardial infarction in patients presenting with ST-segment elevation: The Task Force for the management of acute myocardial infarction in patients presenting with ST-segment elevation of the European Society of Cardiology (ESC). Eur. Heart J..

[5] Naderi SH, Bestwick JP, Wald DS (2012). Adherence to drugs that prevent cardiovascular disease. Am. J. Med..

[6] Spertus JA (2006). Prevalence, predictors, and outcomes of premature discontinuation of thienopyridine therapy afterdrug-eluting stent placement: Results from the PREMIER registry. Circulation.

[7] Kubica A (2015). Discrepancies in assessment of adherence to antiplatelet treatment after myocardial infarction. Pharmacology.

[8] Ho PM (2006). Impact of medication therapy discontinuation on mortality after myocardial infarction. Arch. Int. Med..

[9] Kubica A (2017). Self-reported questionnaires for assessment adherence to treatment in patients with cardiovascular diseases. Med. Res. J..

[10] Tuppin P (2010). Evidence-based pharmacotherapy after myocardial infarction in France: Adherence-associated factors and relationship with 30-month mortality and rehospitalization. Arch. Cardiovasc. Dis..

[11] Chen HY, Saczyński JS, Lapane KL, Kiefe CI, Goldberg RJ (2015). Adherence to evidence-based secondary prevention pharmacotherapy in patients after an acute coronary syndrome: A systematic review. Heart Lung.

[12] Kassab Y, Hassan Y, Abd Aziz N, Ismail O, AbdulRazzaq H (2013). Patients' adherence to secondary prevention pharmacotherapy after acute coronary syndromes. Int. J. Clin. Pharm..

[13] Buszko K (2016). The Adherence Scale in Chronic Diseases (ASCD). The power of knowledge: The key to successful patient—health care provider cooperation. Med. Res. J..

[14] Kubica A (2016). Prediction of high risk of non-adherence to antiplatelet treatment. Kardiol. Pol..

[15] Ho PM (2014). Multifaceted intervention to improve medication adherence and secondary prevention measures after acute coronary syndrome hospital discharge a randomized clinical trial. JAMA Intern. Med..

[16] Zhu B (2011). Factors associated with clopidogrel use, adherence, and persistence in patients with acute coronary syndromes undergoing percutaneous coronary intervention. Curr. Med. Res. Opin..

[17] Bansilal S (2016). Assessing the impact of medication adherence on long-term cardiovascular outcomes. J. Am. Coll. Cardiol..

[18] Choudhry NK (2011). The implications of therapeutic complexity on adherence to cardiovascular medications. Arch. Intern. Med..

[19] Thim T (2014). Clopidogrel discontinuation within the first year after coronary drug-eluting stent implantation: An observational study. BMC Cardiovasc. Disord..

[20] Jánosi A (2017). Adherence to medication after myocardial infarction and its impact on outcome: A registry-based analysis from the Hungarian Myocardial Infarction Registry. Orv Hetil..

[21] Kirchmayer U (2012). Socio-demographic differences in adherence to evidence-based drug therapy after hospital discharge from acute myocardial infarction: A population-based cohort study in Rome, Italy. J. Clin. Pharm. Ther..

[22] Korhonen MJ (2017). Adherence tradeoff to multiple preventive therapies and all-cause mortality after acute myocardial infarction. J. Am. Coll. Cardiol..

[23] Mathews R (2015). Persistence with secondary prevention medications after acute myocardial infarction: Insights from the TRANSLATE-ACS study. Am. Heart J..

[24] Wong MC, Jiang JY, Griffiths SM (2010). Antihypertensive drug adherence among 6408 Chinese patients on angiotensin-converting enzyme inhibitors in Hong Kong: A cohort study. J. Clin. Pharmacol..

[25] Lewey J, Shrank WH, Bowry AD, Kilabuk E, Brennan TA, Choudhry NK (2013). Gender and racial disparities in adherence to statin therapy: A meta-analysis. Am. Heart J..

[26] Reuter H (2015). Long-term medication adherence in patients with ST-elevation myocardial infarction and primary percutaneous coronary intervention. Eur. J. Prev. Cardiol..

[27] Crowley MJ (2015). Medication non-adherence after myocardial infarction: An exploration of modifying factors. J. Gen. Intern. Med..

[28] DegliEsposti L (2012). Adherence to statin treatment and health outcomes in an Italian cohort of newly treated patients: Results from an administrative database analysis. Clin. Ther..

[29] Park YH, Kim H, Jang SN, Koh CK (2013). Predictors of adherence to medication in older Korean patients with hypertension. Eur. J. Cardiovasc. Nurs..

[30] Kang CD (2015). Determinants of medication adherence and blood pressure control among hypertensive patients in Hong Kong: A cross-sectional study. Int. J. Cardiol..

[31] Lacro JP, Dunn LB, Dolder CR, Leckband SG, Jeste DV (2002). Prevalence of and risk factors for medication nonadherence in patients with schizophrenia: A comprehensive review of recent literature. J. Clin. Psychiatry.

[32] Kumbhani DJ (2013). Predictors of adherence to performance measures in patients with acute myocardial infarction. Am. J. Med..

[33] Kriegbaum M, Lau SR (2018). Medication non-adherence and uncertainty: Information-seeking and processing in the Danish LIFESTAT survey. Res. Soc. Adm. Pharm..

[34] Ganasegeran K, Rashid A (2017). The prevalence of medication nonadherence in post-myocardial infarction survivors and its perceived barriers and psychological correlates: A cross-sectional study in a cardiac health facility in Malaysia. Patient Prefer Adher..

